# Indents

**Published:** 1913-02-15

**Authors:** 


### INDENTS.

**☞Brief Articles of Technical or Scientific Value will receive notice and comment. Contributions invited.**

*A New and Positive Method of Cauterizing with Silver Nitrate.* In order to have a rapid and positive effect whenever cauterizing surfaces of decayed dentin or defective spots near the gingival margin, dry the surface, then apply Silver Nitrate, and expose to direct sunlight. At this point use a large magnifying glass to focus the direct rays of sun onto the cauterized surface. It will turn black immediately.—
—W. O. Fellman in *Dental Review.*

*The Use of Cement with Amalgam.* Experiments with amalgam in cavities so made that the fillings when completed could be removed without destroying any part of the cavity surface show that it is extremely difficult to fill a cavity with amalgam that will seal the margins and stop decay. Therefore I consider it good practice to use soft cement in every cavity to be filled with amalgam. I use just enough to cover the cavity surface, removing any surplus with an instrument before starting to use the amalgam, then begin with the amalgam and be extremely careful to pack well, you need not be alarmed at the cement that oozes out at the margins of cavity as it may be removed and the mixture does no harm. This method in my practice gives good service and amalgam fillings may be made that no man need be ashamed of.—
Frank J. Ryan in *Dental Review.*

*In the Cleveland Schools.* Eight out of of every ten pupils in Cleveland public schools are suffering from some physical defect, according to statistics shown at the school medical inspection department, school headquarters, Saturday, which gave the results of the first three weeks of medical inspection in the schools.

Of 3,918 pupils examined during the first three weeks of school, 3,017 were found to be defective. Only 901 were without defects.—*Cleveland Leader*, October 14, 1912.

*Why is a National Dental Association?* The reader may think and likely remark, why the writer does not ask, "Why is a policeman?"

Yet, stop and think a moment. This is not such an idiotic question as it may seem, and judging from the ridiculously small proportion of the profession of the country who attend these annual meetings, the question is eminently pertinent.

Of about forty thousand members of the profession in our country, only about twelve hundred thought it worth their while or were financially able to attend the meeting in Washington last month, and I am constrained to believe that the non-attendance was not due to financial inability with a very large percentage of the profession.

To the writer, it has always seemed a remarkable and deplorable fact that so very few of our profession appreciate the inestimable value derived from association. This unfortunately holds good in local societies as well as in State and National. In trying to fathom the reasons for this condition, the writer has ascribed it to three, viz: Complete indifference as to personal advancement and improvement, a superlative conceit in their own knowledge and ability and utter contempt for that of their fellow practitioners, or selfish fear that by association they might have to part with some of their exclusive, valuable knowledge in order to gain that of others.

It is a deplorable fact that the dental profession is less progressive in this matter of association than any other line of industry. Men in every walk and pursuit of life have discovered the great benefit derived from interchange of thought with their fellows. This has resulted in personal gain and mutual advantage, and increased respect for your fellow and a renewal of personal ambition and endeavor.

There is nothing which will incite ambition to the same extent as to see and know what some one else is accomplishing. And last but not least is the great gain which accrues to the public from this interchange of ideas among men of a calling.

It is a well-known fact that one can not stand still. It must be progress or retrogression. And in these days of lightning advancement and achievement, one may become a "has been" almost over night.

It is a realization of this fact united with a noble ambition, which inspires a few of our profession to form and conduct societies and by an interchange of ideas strive to improve themselves, elevate their profession and benefit mankind. And as a consequence after developing local and State societies a few of our most progressive and ambitious men realized greater possibilities and a larger field, in bringing together in a national organization the brightest and biggest minds of our profession, and recently this idea was advanced still further, and the International Federation was formed, organizing the leading men in our profession of the world, and thus is answered my question, "Why is a National Dental Association?"—A. M. Flood in *Pacific Gazette.*

*Vacuum vs. Compressed Air in Dentistry.* Dr. W. C. Dalbey, of DuQuoin, Ills., in *Dental Review*, June, 1912, suggests the use of a vacuum instead of compressed air to remove the chips, etc., of cavity preparation. By a simple rearrangement of his compressed air apparatus he converted it into a "vacuum cleaner." The compressed air syringe nozzle, somewhat enlarged, answered for a suction nozzle, and by its use, instead of blowing the debris of a nasty carious tooth all over the patient's mouth, and sometimes into his own eyes, it is gathered up and drawn away cleanly. Patients at times complained of an odor from the compressed air, probably caused by oil from the pump; this in the vacuum arrangement is avoided. He finds it satisfactory in removing moisture from a cavity; the blood and saliva from the field of operation when treating cases of pyorrhea; and by placing over the nozzle a small rubber cup, such as is used in polishing teeth, he is enabled to extract from a fistulous opening any pus that may be present. In treating abscess cases he can, by this means draw medicaments placed in the tooth through the fistulous opening. He also finds it an effective and reliable saliva ejector.—*The Dental Brief.*

*Low Fusing Die Metal.*

| | |
|---|---|
| Bismuth | 48 parts. |
| Lead | 19 parts. |
| Cadmium | 13 parts. |
| Tin | 20 parts. |
| | 100 parts. |

*Dr. Ward's Low Fusing Die Metal.*

| | |
|---|---|
| Bismuth | 20 parts. |
| Lead | 8 parts. |
| Cadmium | 4 parts. |
| Tin | 6 parts. |
| | 38 parts. |

*Prevention.* Only four per cent of people die from old age, and five per cent die from accidents, and the rest die from preventable diseases. Most diseases are contagious, some are diseases of ignorance or discretion. If we could do away with the mouth and nose as ports of entry for disease, if we could in other words do away with diseased teeth, tonsils and nasal disorders we could eliminate the majority of the preventable diseases.—Dr. W. A. Evans.

*A Peculiar Case.* A middle-aged widow lady who lived seventeen miles in the country came to me for dental treatment. With no apparent cause she had lost her voice five years before. I found her two lower third molars badly decayed, with the gum tissue growing into the cavities. Though she had not complained of these teeth, she consented, upon my advice, to have these teeth extracted. Within a few minutes after the operation she started on her drive home and before reaching home she was gratefully surprised to find that she could again command the use of her voice.—Dr. G. A. T. in *Digest.*

*Some Observations by Brother Bill.* I had rather have ten clean dollars than ten thousand tainted ones. I am strong for making money, but I regard every dollar made by misrepresentation as a disgrace to the man who made it.

*Courteous Attitude.* If some good fairy would offer me the choice between a fine manner with ordinary mental capacity and great mental capacity with little good manners, I'd take the fine manners assured that it would make me more successful in life than great brains and boorish manners. A genuine courtesy is evidence of love in action, it is the kindly sympathetic thought that takes time to forget itself for a little and really thinks of you and yours. It wins and holds people as nothing else I know of can. To be fine it must be sincere, as the imitation is disgusting. A fine manner is one of the greatest factors in building a practice. Business ability demands that a dentist be clean in person, in body, and in spirit. It demands that he count the costs and use these as a basis for fees. It demands that he acquire the greatest degree of professional skill within his power, and that he add to it the conscience of an honest man, and with these serve his patients. And it demands that every one should have a legitimate profit on his work and that he make judicious use of all profits once they are his.

No, you don't have to be born with business ability. You get it by seeking to reproduce in your own life the business habits you admire in others. You can begin at any time to acquire it, if you are willing to pay the price in patient effort, and you can keep on getting it just as long as your mind is open at the top.

And, in my opinion, the more of it you get, the bigger and happier man and dentist you will be.—*Dental Digest.*

*To Prevent Splint Rivets from Showing in the Anterior Teeth.* After drilling holes through the teeth make a wax splint in the usual way. Force pieces of black lead (lead pencil) through the holes in the tooth and through wax. Remove lead and wax, replace leads in wax, fasten, invest and cast. Remove the leads and countersink holes on lingual side of splint and polish. Countersink deeply on the labial side the holes in the natural teeth. Make a piece of tubing of 22k. plate to fit hole. Cut the tube a little short so that in passing it through the splint and tooth from the lingual side it will not reach through the enamel. It is well to spread the lingual end of the tube a little so that it fits into the countersink in the splint and will not push through. Cement in the usual way and spread tube at both ends until tooth is firm. Then fill the hole in tube with synthetic cement and no gold shows from the front. For riveting, I use an old plate punch remodeled, so that it has two tapering steel points that meet.—Clifford M. Roberts, in *Digest*.

*Prevention of Cancer.* Avoid excessive smoking which predisposes to cancer of the lips, tongue, etc. Inhaling cigarette smoke predisposes cancer of the vocal cords.

Avoid all mechanical irritations of the tongue by rough or irregular teeth, roots, deposits on the teeth, plates, bridges, etc.

Avoid all excessively hot foods, drinks and chemicals irritants which irritate the throat. No food heated to more than 100 degrees F. should be swallowed habitually. Continued use of irritating sprays, washes or lotions of any sort should be avoided.

Avoid very cold drinks and foods in excess especially ices. These are locally bad and also interfere with digestion. Masticate all food thoroughly. Unmasticated food is not easily digested and causes chronic irritation of the entire alimentary tract, which may involve any of the digestive glands or organs.

Don't drink with meals as this tends to bolting the food unmasticated and also interferes with proper digestion. Drink after or between meals. The back teeth should be carefully preserved, free from cavities and irritations of all kinds. Sensitive teeth should be treated, exposed pulps removed and any tooth that can not be filled and freely used and can not be cured should be removed and a proper substitute provided.—Dr. J. F. Little in *British Journal.*

*Effects of Heat on Metals.* H. Chottky in British Dental Journal, December, 1912, records results of experiments in heating metal short of their fusing points. Gold leaf made of pure gold 97 parts, copper 3 parts, when heated to 700 degrees F., becomes wrinkled and shrinks markedly at 1000° F. These changes are less manifest as the plate is made thicker. In hydrogen and nitrogen the changes take place at higher temperature, but no disintegration takes place even at 1500 degrees. He explains these phenomena by saying that the surface tensions in the crystal elements counteract the forces of orientation, which are weakened as the excessive heats are applied. The magnitude of surface tension shows that the ultimate strength per square inch of surface is less in thin than thick sheets. It is claimed that in mechanically deformed metals, surface tensions are so concentrated as to produce recrystalization in proportion to their magnitude.

*Red Rubber Poisoning.* J. W. Dent in British Dental Journal for December, 1912, relates a case of successful substitution of red vulcanite plates with black vulcanite for the cure of a distressing case of a swollen tongue, throat and severe stomach disorder; there was no ulceration or evidence of salivation. The patient had worn the plates twelve years and had suffered from the swelling and redness of the tissues for several years and taken treatment from physicians without benefit. After the new black vulcanite plates were inserted there was more or less numbness and insensibility for several weeks, but this gradually disappeared and the patient is now free from all disease and annoyance.

*Elongation of Posterior Teeth in Partial Vulcanite Plates.* In reply to C. B. M., November *Brief*, permit me to suggest several probable causes. It is a trouble of which I have had, and still have, more than I like. Recognition of possible causes assists in making the trouble less, as it suggests where special care is needed, nevertheless, in spite of all care it will now and again occur "in the best regulated dental laboratories." Presuming that due care has been taken when testing the occlusion and when closing the flask, it may occur from undue force in packing, pressing the teeth deeper in the plaster, especially if dry heat has been used. While a moderate degree of dry heat hardens newly set plaster, an excess of heat has the contrary effect. One may unwittingly overheat a flask by placing it over a gas flame or other like arrangement where all the heat is applied to the bottom of the flask. Again, it is quite possible for a trial plate to adapt itself to the form of the mouth, in the mouth, a little different than it does upon the model; the difference may be slight, it may effect all the teeth and yet be noticeable only on the back teeth, as a slight lengthening there holds the jaws apart while the same increase in length of the non-occluding teeth would not be noticed. It is safer to allow plenty of time for the plaster to harden before attempting to pack, and also safer to use steam heat for softening the rubber when closing the flask rather than dry heat. This may readily be done by placing a small quantity of water in the vulcanizer, placing the flask in the vulcanizer, elevated so as not to be in the water, and heating it up with the cover laid in place but not screwed down. On several occasions when lengthening of the teeth has occurred I have attributed it to oversight in leaving the flask under heat too long, a slight vulcanizing having taken place that rendered the rubber less plastic.

There is a happy mean between commencing to screw down the flask too soon and waiting too long. It is well to also remember that heating the vulcanizer too rapidly will tend to the formation of certain gases that expand the rubber with considerable force, usually producing a degree of porosity, but at times so slight that it will not show in the finished work, and yet it may slightly displace the teeth. In the early days of vulcanite work it was thought necessary to overpack the flask under the impression that by so doing the vulcanite was rendered more dense and stronger. More extended experience proved this to be a mistake; there was no improvement in the product, and much mischief done. Broken blocks, elongated teeth and misfits from crushed models were quite common. Since we have learned that just a little more than enough is quite enough, and to open the flask and inspect its contents during the process of packing, these misfortunes are infrequent, nevertheless, now and again the grindstone is needed to reduce the length of molars and occasionally bicuspids, more frequently in partial cases because they must permit the remaining natural teeth to occlude while a slight lengthening in a full denture may not be a serious defect.—M. B. S. in *Dental Brief.*

*The Digestibility and Nutritional Value of Cheese.* At a period when so much painstaking research is being expended upon nutritional problems and the influence of diet upon health and longevity, the results of the investigation of the U. S. Department of Agriculture regarding the nutritional and economic value of cheese as recorded in bulletins issued by the Department are of great interest and value.

The most recent of these bulletins, Cheese and its Economical Uses in the Diet (February 26, 1912), contains, in addition to an interesting account of the process of cheese making and a description of the characteristic qualities of all the better known varieries of the product, foreign and domestic, a large number of recipes in which cheese can be employed as an agreeable and nutritional adjunct, or of which it forms the chief ingredient.

In an earlier bulletin (February 25, 1911), the Department published the results of its experimental investigation of the digestibility of cheese, there being quite a widespread belief that while as a condiment or appetizer it may aid digestion; it is itself not very digestible, and is especially prone to produce constipation; a popular misconception which has been perpetuated in the adage, "Cheese the surly elf, digest all things except itself."

On this point the conclusion of the investigators is that "cheese properly prepared and used is not generally a cause of physiological disturbances, and that it may easily be introduced into the bill of fare in such quantities as to serve as the chief source of nitrogenous food, and may be made a substitute for other nitrogenous foods when such substitution is desired."

As is well known, cheese with bread forms an important part of the food supply of the Swiss, a sturdy, healthy race, and also of the working classes of many other nations of Europe; while in America it occupies a place entirely secondary to meat in its various forms. The conclusion of C. F. Doane, who conducted the investigation, reported in the Agricultural Department bulletin of 1911, is that:

"Cheese can no longer be discriminated against because of a suspicion that it is not healthful food. The absolute lack of any disturbance of the general health of the subjects used in the experiments reported in this bulletin is proof that cheese can be eaten in large quantities without danger to health. The Swiss cheesemakers, also many of the Swiss farmers of southern Wisconsin, eat unusually large quantities of cheese, and they are noted for athletic attainments and physical endurance. They brought the custom of eating cheese from their native country, where cheese is a very important item in the diet. The consuming public, especially that part of it which needs to practice economy in buying foods, would do well to turn its attention a little more toward cheese, since greater quantities can be used at a saving to the consumer."

In view of the dimished supply and increased cost of flesh foods in this and many other countries, these conclusions have an economic as well as a physiologic interest and value; and there can be but little doubt that from every point of view more cheese and less meat would be an advantageous change in the dietary of the average American.

Much of the alleged indigestibility of cheese is doubtless due to the custom of eating it, not as an integral part of the daily ration, but as an addition to an already sufficient, or more than sufficient meal; with the results which naturally attend overfeeding. Also contributory to ill-effects is its deglutition in masses difficult of penetration by the gastro-intestinal secretions. If eaten at the proper time, in proper quantity and properly chewed and insalivated, good, plain dairy cheese will be found to be readily digestible as well as highly nutritive food material.—December *Dental Brief.*

*Dental Recognition.* It is human nature for us to feel that we do not get all to which we are entitled, but perhaps it would be as nearly true to say that we receive about what we deserve. We are all clamoring for recognition, or keep secretly, wondering why our merits and attainments are not appreciated and fail to receive their fitting reward.

We are a race of self-centered egotists, the base of all things being the spot where our own feet are planted. And it is wisely ordained that it should be so. This supreme consciousness of self is the source of divine unrest, which spurs each to sustained effort, to the end of securing greater rewards.

A profession is but an aggregation of individuals with a common bond that consists in mutual work and aims. The psychology of the average man in a given profession is the psychology of the profession itself. The man who thinks deeply may not always think broadly; concentration of effort is intensive rather than diffusive. And it is this that makes members of any profession self-centered, often leading to a magnifying of their own importance in the scheme of things, to the detriment of the other millions who are absorbed with other lines of thought and endeavor.

We become restless at the scant attention the profession of dentistry attracts, forgetting that to the great mass of men and women any appeal must contain a promise of material comfort or relief from present discomfort or pain.

Doubtless, like men of other callings, individually and collectively, we receive about the recognition that we deserve. It does not flatter our vanity to admit this, but is a wholesome fact to get before us.

For some reason, the dentist is a super-sensitive being. Oftentimes he assumes the attitude of inviting and expecting ill treatment, and anyone with this state of mind is rarely disappointed. If a cub reporter writes up a dental convention flippantly, the dentist persuades himself that it is but a reflection of the low estimate in which the public holds this great profession. He is wounded if his patients ask him how much an operation will cost, just as they would ask a watchmaker the cost of putting a timepiece in repair; and he demands that his services shall be regarded as strictly professional, the discussion of fees to be left in the background.

But the task of training the great public—a public not reared in an atmosphere of dentistry—is too great to be accomplished in a day. The results will always be greater if the dentist will set about improving himself, and if the profession will keep its attention centered on a better, fuller service to humanity.

As we have previously hinted, dentistry has done remarkably well. As a branch of the healing art it is extremely modern. Its present high standing hasn't been won by a beating of tom-toms. It hasn't gained its respectable proportions by artificial feeding. Its recognition has been won by the tens of thousands of industrious, capable men throughout the world, who, day by day, have performed their tasks of alleviating pain and repairing defects in the oral cavity. Had their work been less efficient, dentistry would now stand lower in public esteem; had it been even more efficient, our profession to-day would be more highly regarded.

We must not deceive ourselves; if we would achieve, we must have merit—and then more merit.

The individual dentist, impatient to get on and possibly ill-equipped may resort to artificial methods in order to win recognition and get money. To get results quickly he may have recourse to lurid advertising, justifying himself on the ground that he is entitled to live and pay his office rent. There isn't anything fundamentally immoral in his method; but it is in such questionable taste that were this practice to become the rule, dentistry would soon cease to be one of the learned professions. His attempt at justification lies in the fact that the business man advertises that which he has to sell, and the professional man may do the same. But the distinction lies just here: The merchant has goods to sell; the dentist has his service to offer. One is impersonal; the other personal. There is a vast difference between dickering over a barrel of sugar and proclaiming your own personal skill and efficiency.

Then, too, the advertising dentist takes an unfair advantage of his ethical brother. He succeeds through advertising, because most dentists do not advertise. If all resorted to printers' ink, a vast expense would be added to dental practice, which all would share, with possibly no increase in practice to any. Another form of advertising consists in elaborate, often garish, equipment. Of course, nobody can be too well equipped, but many are over equipped. Utility and taste should govern the professional man's surroundings. But elaborate appliances and ornate furnishings rarely produce a satisfactory practice and never help to give the profession higher standing.

The old-fashioned philosophy that the world will recognize us in proportion to what we are and what we do, yet holds good. But whatever may be true of the individual, as a profession we shall be known by our fruits. And this does not mean that we shall be judged alone by those eminent specialists whom we delight to honor, but by the tens of thousands of earnest men, scattered throughout the land, who are quietly and conscientiously doing their work in every city, village and hamlet, whom their patients recognize as good citizens and men of skill in their calling.—Dr. Litch in *Dental Brief*.

*Our Work.* Only occasionally, when we stop to think, do we realize what a lot of genuine fun we can get out of our work. Of course every one can readily see the pleasure that artists of all kinds take in their pursuits—the pleasure a painter gets from his picture, the sculptor from his statue, the composer when the band crashes out the stirring music that is the fruit of his toil. But what we are more directly concerned with right here is the pleasure the every day man can get out of his work—the farmer from tilling the soil, the business man in closing a deal, you—in keeping your work going at top-notch efficiency.

When the philosopher said, "So much as you put into your work, so much it will return to you," he was not trying to put a lot of meaningless high-brow stuff over on us. Any man who has done a day's honest work knows the remarkable simplicity and the vital truth of that saying. The better you do your work, the better you like it—the better satisfied you are with your position and with yourself. Doing our work with interest, working heartily, cheerfully, steadfastly, and giving it the best that is in us robs our toil of a great deal of the laboriousness it may contain.—The Sales Manager, in *American Machinist*.

